# Sympathetic Nervous System Control of Carbon Tetrachloride-Induced Oxidative Stress in Liver through *α*-Adrenergic Signaling

**DOI:** 10.1155/2016/3190617

**Published:** 2015-12-21

**Authors:** Jung-Chun Lin, Yi-Jen Peng, Shih-Yu Wang, Mei-Ju Lai, Ton-Ho Young, Donald M. Salter, Herng-Sheng Lee

**Affiliations:** ^1^Graduate Institute of Medical Sciences, National Defense Medical Center and Department of Pathology, Tri-Service General Hospital, 325 Section 2, Cheng-gong Road, Neihu, Taipei 114, Taiwan; ^2^Division of Gastroenterology and Hepatology, Department of Internal Medicine, Tri-Service General Hospital, National Defense Medical Center, Taipei, Taiwan; ^3^Department of Pathology, Tri-Service General Hospital, National Defense Medical Center, Taipei, Taiwan; ^4^Department of Pathology and Laboratory Medicine, Kaohsiung Veterans General Hospital, No. 386, Dazhong 1st Road, Zuoying District, Kaohsiung 81362, Taiwan; ^5^Division of Gastroenterology, Department of Internal Medicine, Cardinal Tien Hospital, Fu Jen Catholic University, Taipei, Taiwan; ^6^Center for Molecular Medicine, MRC IGMM, University of Edinburgh, Edinburgh, UK

## Abstract

In addition to being the primary organ involved in redox cycling, the liver is one of the most highly innervated tissues in mammals. The interaction between hepatocytes and sympathetic, parasympathetic, and peptidergic nerve fibers through a variety of neurotransmitters and signaling pathways is recognized as being important in the regulation of hepatocyte function, liver regeneration, and hepatic fibrosis. However, less is known regarding the role of the sympathetic nervous system (SNS) in modulating the hepatic response to oxidative stress. Our aim was to investigate the role of the SNS in healthy and oxidatively stressed liver parenchyma. Mice treated with 6-hydroxydopamine hydrobromide were used to realize chemical sympathectomy. Carbon tetrachloride (CCl_4_) injection was used to induce oxidative liver injury. Sympathectomized animals were protected from CCl_4_ induced hepatic lipid peroxidation-mediated cytotoxicity and genotoxicity as assessed by 4-hydroxy-2-nonenal levels, morphological features of cell damage, and DNA oxidative damage. Furthermore, sympathectomy modulated hepatic inflammatory response induced by CCl_4_-mediated lipid peroxidation. CCl_4_ induced lipid peroxidation and hepatotoxicity were suppressed by administration of an *α*-adrenergic antagonist. We conclude that the SNS provides a permissive microenvironment for hepatic oxidative stress indicating the possibility that targeting the hepatic *α*-adrenergic signaling could be a viable strategy for improving outcomes in patients with acute hepatic injury.

## 1. Introduction

The liver is one of the most highly innervated tissues in mammals. The interaction between hepatocytes and sympathetic, parasympathetic, and peptidergic nerve fibers through a variety of neurotransmitters and signaling pathways is recognized as being important in the regulation of hepatocyte function and hepatic response to injury [1]. Studies of hepatic architecture identify adrenergic nerve fibers extending from perivascular plexus in the portal space into the lobule [2]. Sympathetic nervous system (SNS) transmission to hepatocytes occurs through release of norepinephrine and epinephrine as neurotransmitters from intrahepatic nerve endings and by delivery as hormones from adrenal glands. In addition to being important for a range of functions such as regulating hepatic circulation, metabolism, and bile formation the SNS is also known to modulate both liver regeneration and fibrosis [3–5].

Potential associations between the SNS and oxidative stress are indicated from previous studies. Hepatic monoamine oxidases catalyze oxidative deamination of catecholamines such as norepinephrine and epinephrine. During this process hydrogen peroxide (H_2_O_2_) is generated and further converted to water by glutathione peroxidase during which glutathione is utilized [6]. Thus, oxidation of catecholamines is a source of reactive oxygen species (ROS) [7]. Long-term elevation of epinephrine can deplete hepatic glutathione as epinephrine both decreases the rate of glutathione synthesis in the liver and increases the rate of glutathione release from the liver whilst decreasing the rate of recycling of oxidized glutathione [8–10]. Similarly, epinephrine stimulates H_2_O_2_ production via cyclic 3′-5′-adenosine monophosphates in macrophages [11]. Intense physical work is known to increase sympathetic activity and ROS production in the rodent heart [12]. The knowledge that *β*-adrenergic stimulation is the main driver of ROS generation in mitochondria [13] has indicated the use of *β*-adrenergic receptor blockers to reduce oxidative stress in cardiac failure [14, 15].

Although these studies clearly suggest a role for catecholamines in the modulation of oxidative stress, whether the SNS affects oxidative stress in the liver has yet to be established. CCl_4_ is a classic model compound for inducing free radical damage in the liver [16, 17], being metabolized to form trichloromethyl and trichloromethyl peroxy radicals which covalently bind to proteins, lipids, and nucleic acids to initiate lipid peroxidation, generate 4-hydroxy-2-nonenal (4-HNE), and thus induce liver damage [17]. This model is also useful for characterization of xenobiotic-induced hepatotoxicity, screening of hepatoprotective effects of drugs, and studying mechanisms of human liver injury [18, 19]. An ablation of the SNS exerts a protective effect against CCl_4_ induced acute liver injury in mice [20]. In the current study peripheral injection of the neurotoxin 6-hydroxydopamine hydrobromide (6-OHDA) was used to induce chemical sympathectomy and the effects on acute CCl_4_ induced hepatic lipid peroxidation were assessed. We also tested the hypothesis that adrenergic signaling is required for control of the oxidative stress in hepatocytes after acute CCl_4_ exposure. The observation that chemical sympathectomy or treatment with the *α*-adrenoreceptor antagonist has profound inhibitory effects on CCl_4_ induced hepatic oxidative injury has important implications for understanding of how the response to liver injury may be controlled and, on a clinically applicable basis, indicate potential novel strategies for management of acute or, indeed, chronic liver injury.

## 2. Materials and Methods

### 2.1. Animal Care

Male C57Bl/6JNarl mice aged 8 weeks were obtained from the National Laboratory Animal Center in Taipei, Taiwan. They were maintained under controlled conditions (22 ± 1°C and 12 h day/night rhythm) and fed standard laboratory food.

### 2.2. Chemical Sympathectomy and Antagonists

Mice were intraperitoneally injected with 100 mg kg^−1^ 6-OHDA (Sigma, St. Louis, MO, USA) in 0.1% ascorbic acid (Sigma) in phosphate buffered saline (PBS, Gibco, Gaithersburg, MD) daily over 5 consecutive days. Peripheral administration of 6-OHDA results in a “chemical sympathectomy” by depleting sympathetic fibers [20] and has previously been found to induce an 85% decrease in norepinephrine levels in the liver [21]. Control mice received injections of 0.1% ascorbic acid in PBS. One day following the final treatment with 6-OHDA, mice were subjected to the acute oxidative liver injury protocol described below. Phentolamine (10 mg kg^−1^) and nadolol (20 mg kg^−1^) were injected in 0.9% saline daily intraperitoneally, commencing 5 days before CCl_4_ administration, while control mice were injected with 0.9% saline vehicle.

### 2.3. Acute Oxidative Liver Injury Induced by CCl_4_

Mice were injected intraperitoneally with a single dose of CCl_4_ (Sigma) (12.5% in olive oil (Sigma), 2 mL kg^−1^). Control groups were treated with vehicle (2 mL kg^−1^ of olive oil). At 24 h after CCl_4_ or vehicle treatment, mice were weighed and then euthanized by carbon dioxide asphyxiation. Blood was collected from the heart for analysis of serum AST, ALT, LDH, and ALP by standard enzymatic methods. The liver was then removed, weighed, and processed for further analysis as described.

### 2.4. Immunostaining and Assessment of Hepatocyte Nuclear Morphology

Samples of liver from mice with or without chemical sympathectomy were embedded in optimum cutting temperature (OCT) (Tissue-Tek, Sakura, CA, USA) compound and quickly frozen. Five *μ*m thick sections were cut and fixed with methanol/acetone. Immunohistochemistry for identification of sympathetic nerve fibers was performed with a primary antibody against tyrosine hydroxylase (the key enzyme for norepinephrine production in sympathetic nerve endings, Millipore, Billerica, MA, USA) and an Alexa 488 conjugated secondary antibody (against rabbit IgG, Invitrogen Corporation, Carlsbad, CA, USA). Following a final incubation for 1 h with propidium iodide (50 *μ*g/mL) (Invitrogen) sections were viewed with a Zeiss LSM 510 Meta inverted confocal microscope using a LD-Achroplan 20x lens.

For assessment of nuclear morphology sections were washed twice in TBST (12.5 mM Tris/HCl, pH 7.6, 137 mM NaCl, and 0.1% Tween 20) before 4′,6-diamidino-2-phenylindole dihydrochloride (DAPI) (Sigma) diluted 1 : 10,000 in PBS for 5 min at room temperature. Slides were mounted with glycerin, coverslipped, and examined using a fluorescence microscope (Olympus BX-51).

### 2.5. Histological Analysis of the Liver

The liver left lateral lobes were removed and fixed in 10% buffered formalin before standard tissue processing, embedding in paraffin wax, and cutting into 5 *μ*m sections which were stained with hematoxylin and eosin. Histological examination was performed in a blinded fashion by three experienced pathologists. The severity of hepatic injury was scored as described by Camargo et al. [22] according to the following criteria: 0, minimal or no evidence of injury; 1, mild injury with cytoplasmic vacuolization and focal nuclear pyknosis; 2, moderate to severe injury with extensive nuclear pyknosis, cytoplasmic hypereosinophilia, and loss of intercellular borders; and 3, severe necrosis with disintegration of hepatic cords, hemorrhage, and neutrophil infiltration. Areas of hepatocyte necrosis were expressed as the percentage of damaged liver architecture, measured in 10 randomly selected high-power fields per section using ImageJ software (version 1.48u, US National Institutes of Health).

### 2.6. Estimation of Hepatic Lipid Peroxidation Assay

Samples of liver were homogenized on ice in PBS containing butylhydroxytoluene and centrifuged at 10,000 g (4°C, 5 min). 4-HNE adducts were measured in supernatants with the OxiSelect HNE-His adduct enzyme-linked immunosorbent assay (ELISA) kit (Cell Biolabs, Inc., San Diego, CA) in accordance with the manufacturers' instructions. The concentration of 4-HNE adducts was normalized per microgram of total protein, the concentration of the latter being determined by the Lowry method.

### 2.7. Transmission Electron Microscopy

Liver tissues were fixed in a mixture of 4% paraformaldehyde and 0.5% glutaraldehyde in PBS, pH 7.4, and prepared routinely for transmission electron microscopy with final embedding in LR white resin. Ultrathin sections (70–80 nm) were cut, placed on a nickel grid, and then examined under a Hitachi H-600 transmission electron microscope.

### 2.8. 8-Hydroxy-2-deoxyguanosine (8-OHdG) Assay

Total DNA from liver was extracted using the Qiagen DNeasy blood and tissue kit according to the manufacturer's instructions (Qiagen, Valencia, CA, USA). A NanoDrop 1000 spectrophotometer (Thermo Fisher, Pittsburgh, PA, USA) was utilized to measure DNA purity. Briefly, pH of DNA (25 *μ*g) was adjusted to 5.2 with 3 M sodium acetate. The DNA reaction mixture was subjected to 1 *μ*L of nuclease P1 (1 U/*μ*L) digestion for 2 h at 37°C. After 2 h of incubation, 15 *μ*L of 1 M Tris-HCl (pH 8.0) was used to bring the pH back to 7.4, followed by treatment with 5 *μ*L of alkaline phosphatase (1 U/*μ*L stock) for 1 h. The reaction mixture was centrifuged for 5 min at 6,000 g, and the supernatant was collected for the 8-OHdG assay using the OxiSelect oxidative DNA damage ELISA kit (Cell Biolabs, Inc., San Diego, CA, USA) according to the manufacturer's instructions. Known standards were also included in the assay to allow accurate quantitation. Six livers were used for each experimental group.

### 2.9. Cytokines/Chemokines Antibody Array

Livers were homogenized in lysis buffer (kit component) and then centrifuged to collect the supernatant to detect 40 cytokines/chemokines on the RayBio Mouse Inflammation Antibody Array 1 membrane (RayBiotech, Inc., Norcross, GA, USA), according to the manufacturer's protocol. We pooled six samples per group to obtain 300 *μ*g total proteins per membrane (one membrane for each group). The membranes were blocked in 2 mL of blocking buffer for 30 min and then incubated with pooled supernatant at room temperature for 2 h. The samples were then decanted from each container, and the membranes were washed three times with 2 mL of wash buffer I, followed by two washes with 2 mL of 1x wash buffer II at room temperature with shaking. The membranes were incubated in 1 : 250-diluted biotin-conjugated primary antibodies at room temperature for 2 h and washed as above before incubation in 1 : 1000-diluted horseradish peroxidase-conjugated streptavidin. After incubation in horseradish peroxidase-conjugated streptavidin for 1 h, the membranes were washed thoroughly and exposed to a peroxidase substrate for 5 min in the dark before imaging. The membranes were scanned and analyzed by using TotalLab Quant software (TotalLab Ltd., Newcastle upon Tyne, UK). Proteins with >2-fold differences in their expression levels between the saline + CCl_4_ group and the saline + olive oil group were considered as differentially expressed.

### 2.10. Statistical Analysis

The results were expressed as mean ± standard deviation (SD). Results were analyzed using Student's *t*-test for unpaired data. The statistical significance level was a *p* value of < 0.05.

## 3. Results

### 3.1. Alleviation Effect of Chemical Sympathectomy on Lipid Peroxidation in CCl_4_ Induced Hepatic Injury

Tyrosine hydroxylase-expressing fibers were readily identified in the border of periportal areas of saline-treated mice but were essentially absent in 6-OHDA-treated animals (Supplementary Data, Figure S1 in Supplementary Material available online at http://dx.doi.org/10.1155/2016/3190617) confirming successful hepatic sympathectomy. There was no hepatic necrosis in saline- or 6-OHDA-treated mice treated with olive oil alone. The extent of CCl_4_ induced hepatic necrosis was significantly greater in saline-treated mice than in 6-OHDA-treated mice (32.1 ± 8.6% versus 9.2 ± 4.4%, *p* = 0.0007) (Supplementary Data, Figures S2(a) and S2(b)). Similarly the severity score of hepatic injury was significantly greater (Supplementary Data, Figure S2(c), *p* < 0.0001) in the saline + CCl_4_ group (2.7 ± 0.5) when compared to the 6-OHDA + CCl_4_ group (1.5 ± 0.5). Biochemical markers of liver injury, including aspartate aminotransferase (AST; 4,115 ± 1,755 versus 17, 830 ± 3, 078 IU/L, *p* < 0.0001), alanine aminotransferase (ALT; 7,809 ± 2,527 versus 15,519 ± 4,678, *p* = 0.0052), alkaline phosphatase (ALP; 56 ± 26 versus 128 ± 11, *p* = 0.0013), and lactate dehydrogenase (LDH; 11,139 ± 5,496 versus 28,764 ± 8,063, *p* = 0.0001) levels 24 h after CCl_4_ treatment were lower in serum from 6-OHDA-treated mice compared to the saline-treated group (Supplementary Data, Table S1).

As secondary products during lipid peroxidation, malondialdehyde appears to be the most mutagenic product of lipid peroxidation, whereas 4-HNE is the most toxic and considered as “one of major generators of oxidative stress” and a “major lipid peroxidation product” [23–25]. Thus, we investigated ROS-induced lipid damage as measured by changes in 4-HNE levels in the livers of saline- and 6-OHDA-treated mice 24 h after CCl_4_ administration (Table 1). As expected, 4-HNE was elevated in the livers of saline-treated mice 24 h after CCl_4_ treatment (*p* = 0.0122). This increase in lipid peroxidation was blocked when mice were treated with 6-OHDA prior to CCl_4_ (*p* = 0.0053).

### 3.2. Protective Effect of Chemical Sympathectomy on the Ultrastructure of Hepatocyte Injury

**Figure 1:**
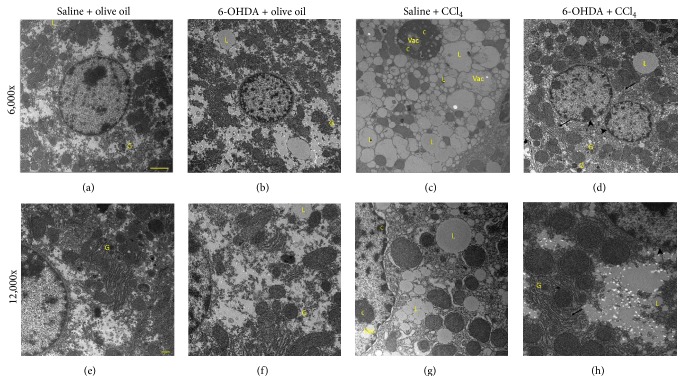
Effect of 6-OHDA on CCl_4_ induced changes on hepatocyte morphology. Representative micrographs of transmission electron microscopy (magnification 6,000x or 12,000x) in the liver tissues. (a and e) The saline + olive oil group; (b and f) the 6-OHDA + olive oil group; (c and g) the saline + CCl_4_ group; and (d and h) the 6-OHDA + CCl_4_ group. Arrows denote rough endoplasmic reticulum. Arrowheads denote perinuclear space. C, chromatin; G, glycogen deposits; L, lipid drops; Vac, vacuolization. Scale bar denotes 100 nm in (a)–(d) and 500 nm in (e)–(h).

Elevation in lipid peroxidation levels has been reported to correlate with ultrastructural changes in hepatocytes following CCl_4_ exposure since 4-HNE can promote organelle damage [25–27]. In the current study the ultrastructure of hepatocytes was normal in the olive oil-treated groups (Figures 1(a), 1(b), 1(e), and 1(f)). Twenty-four hours after CCl_4_ administration, ultrastructural changes including expansion of the perinuclear space with a pyknotic condensed nucleus, increased number and size of lipid globules, glycogen loss, and mitochondrial swelling with loss of cristae in addition to dilation of the cisternae of rough endoplasmic reticulum were evident (Figures 1(c) and 1(g)). In comparison to the saline + CCl_4_ mice, the number and size of lipid globules were decreased and glycogen deposits were readily identifiable within hepatocytes in the 6-OHDA + CCl_4_ group. Similarly the organelle and cytoplasmic structures appeared preserved from the deleterious effects of CCl_4_ (Figures 1(d) and 1(h)).

### 3.3. Attenuation Effect of Chemical Sympathectomy on Hepatic Nuclear Damage

**Figure 2:**
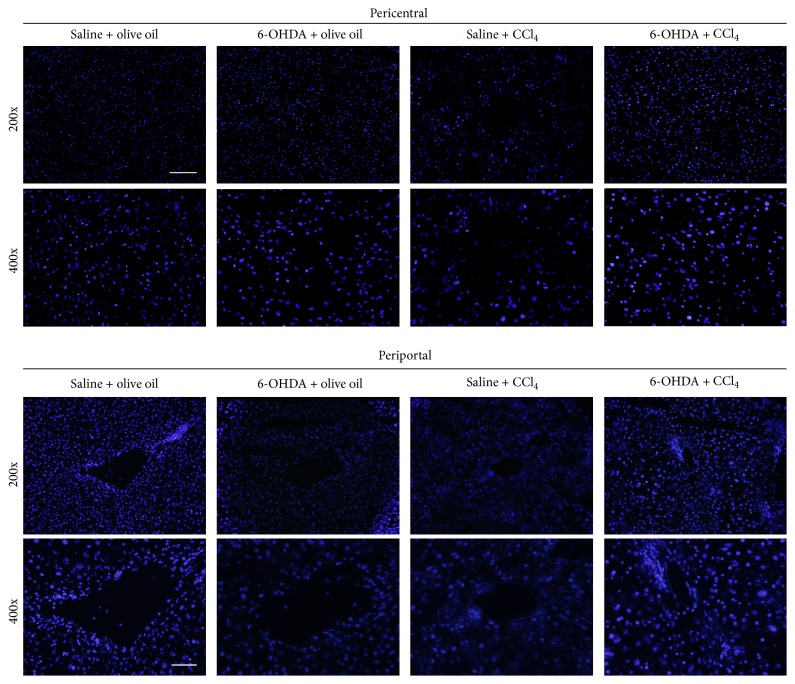
Effect of 6-OHDA on CCl_4_ induced changes in hepatocyte nuclei. Representative micrographs of DAPI stained nuclei (magnification 200x or 400x) in the liver tissues. Typical images were selected from each experimental group (original magnification 200x or 400x). Scale bar = 100 *μ*m in 200x and 50 *μ*m in 400x.

Lipid peroxidation products, in particular, 4-HNE, are known to promote hepatocyte nuclear loss or injury [24, 27, 28] which can be visualized by DAPI staining [29]. The nuclei in hepatocytes of olive oil alone-treated mice were round and emitted even blue fluorescence (Figure 2). In the necrotic pericentral region livers of saline-treated mice 24 h after CCl_4_ administration nuclei were absent, condensed, or fragmented. 6-OHDA effectively preserved the integrity and morphology of hepatic nuclei in CCl_4_-treated mice (Figure 2).

### 3.4. Inhibition Action of Chemical Sympathectomy on Oxidative DNA Damage in Liver

**Figure 3:**
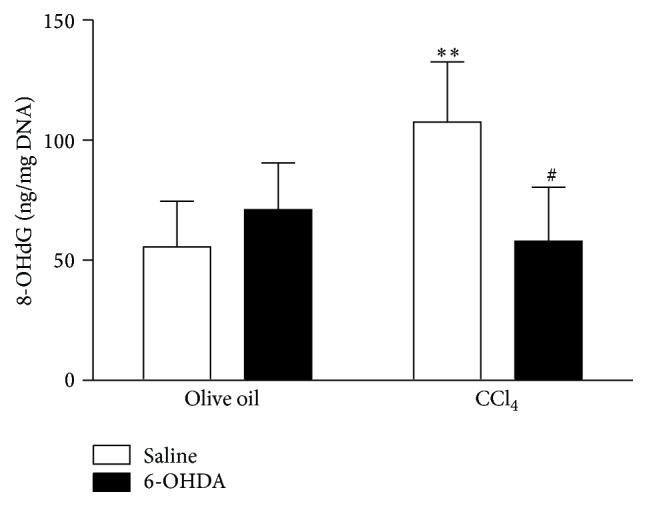
Effect of 6-OHDA on CCl_4_ induced oxidative DNA damage. Oxidized DNA in liver was measured 24 hours after exposure to olive oil or CCl_4_ following pretreatment with saline of 6-OHDA. Data plotted are mean and SD (*n* = 6 animals in each group).  *∗∗* denotes significant differences (*p* < 0.01) compared with the saline + olive oil group.  # denotes significant differences (*p* < 0.05) compared with the saline + CCl_4_ group.

Since 4-HNE also has high capability of reaction with DNA to cause DNA damage [25, 30], we examined whether sympathectomy attenuates liver oxidative DNA damage by analyzing 8-OHdG content in hepatocyte. The level of 8-OHdG in CCl_4_-treated livers without sympathectomy was increased by 94% when compared with that in olive oil-treated livers without sympathectomy (Figure 3, *p* = 0.0092). Pretreatment with 6-OHDA, on the other hand, abrogated this increase (*p* = 0.0113). There was no significant difference in levels of 8-OHdG between the 6-OHDA + olive oil group and saline + olive oil group (Figure 3).

### 3.5. Analysis of Cytokine and Chemokine Protein Profiling in Livers

**Figure 4:**
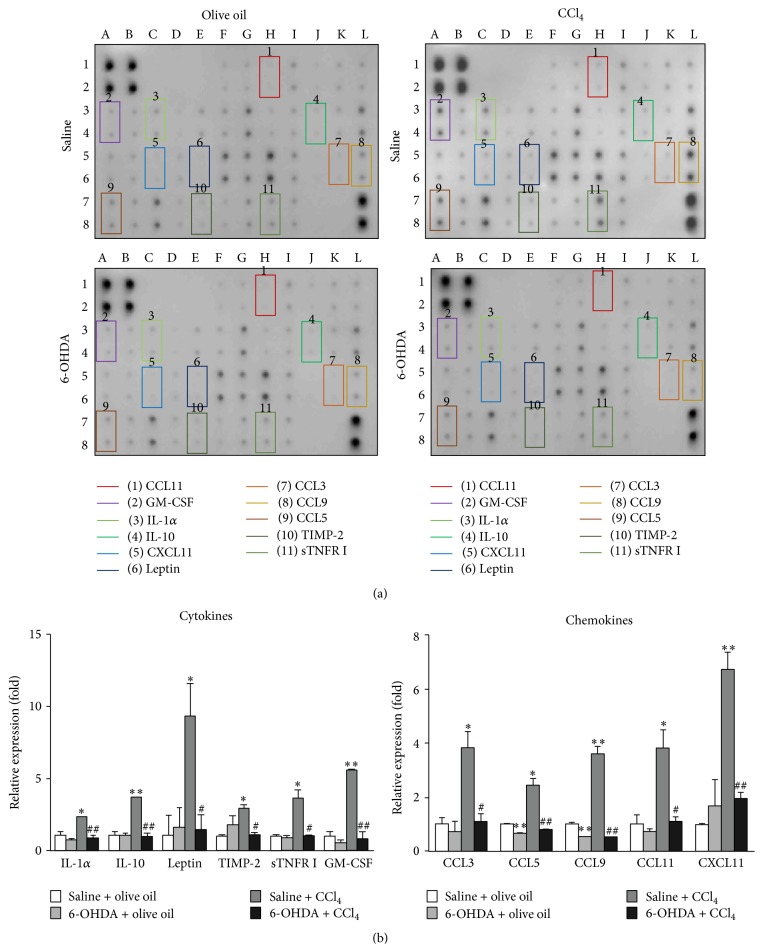
Effect of 6-OHDA on levels of hepatic cytokines and chemokines in the absence and following CCl_4_ treatment. (a) A cytokine array assay in the liver of the saline + olive oil group, the 6-OHDA + olive oil group, the saline + CCl_4_ group, and the 6-OHDA + CCl_4_ group was measured. Altered cytokines (twofold or more), including IL-1*α*, IL-10, leptin, TIMP-2, sTNFR I, GM-CSF, CCL3, CCL5, CCL9, CCL11, and CXCL11, are indicated by boxes. (b) The relative density of cytokines and chemokines was normalized with the internal control and expressed as a ratio of the expression level of cytokines and chemokines in each group divided by the expression level in the saline + olive oil group. Each value represents the average of two replicated spots on the membrane. In all figures,  *∗* denotes significant differences compared with the saline + olive oil group (*p* < 0.05). *∗∗* denotes significant differences compared with the saline + olive oil group (*p* < 0.01).  # denotes significant differences compared with the saline + CCl_4_ group (*p* < 0.05).  ## denotes significant differences compared with the saline + CCl_4_ group (*p* < 0.01).

It is known that 4-HNE plays a role as mediator of inflammatory processes with interactions with the cytokine networks [31–33]. In the current study we investigated the inflammatory response using a multiplex of 40 cytokines and chemokines. The results are shown in Figure 4. Following administration of CCl_4_ there was increased expression of a number of cytokines including IL-1*α* (2.28-fold, *p* = 0.0192), IL-10 (3.64-fold, *p* = 0.0070), leptin (9.27-fold, *p* = 0.0496), tissue inhibitor of metalloproteinase-2 (TIMP-2, 2.90-fold, *p* = 0.0087), soluble tumor necrosis factor receptor I (sTNFR I, 3.62-fold, *p* = 0.0228), and granulocyte-macrophage colony-stimulating factor (GM-CSF, 5.58-fold, *p* = 0.0019). There was also an increase in expression of 5 inflammation-associated chemokines. These were CCL3 (3.80-fold, *p* = 0.0243), CCL5 (2.42-fold, *p* = 0.0153), CCL9 (3.57-fold, *p* = 0.0064), CCL11 (3.79-fold, *p* = 0.0344), and CXCL11 (6.71-fold, *p* = 0.0057). With chemical sympathectomy, in the absence of CCl_4_, there was reduction of protein levels of CCL5 (0.66-fold, *p* = 0.0022) and CCL9 (0.54-fold, *p* = 0.0083) when compared with the saline + olive oil group but no significant change in the levels of other cytokines and chemokines was evident. Pretreatment with 6-OHDA inhibited the increase in hepatic cytokine and chemokine levels induced by CCl_4_.

### 3.6. Hepatoprotective and Antioxidant Effects of *α*-Adrenergic Blockade against CCl_4_ Induced Oxidative Damage

**Figure 5:**
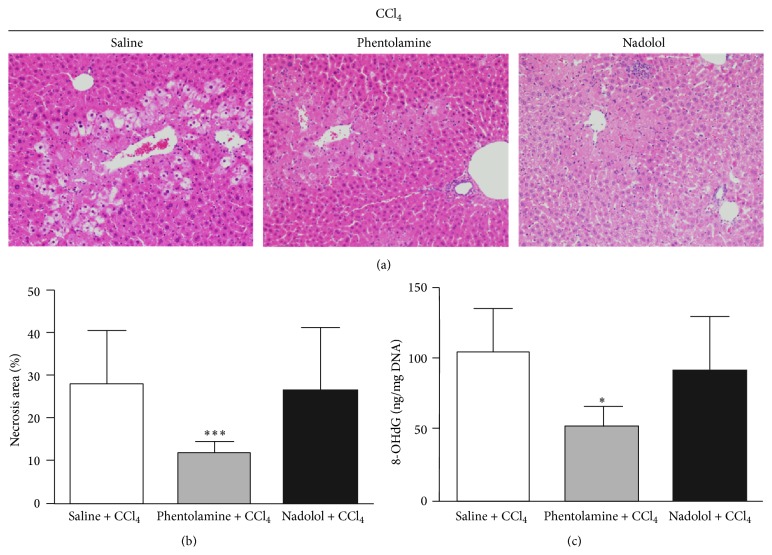
Effect of *α*- or *β*-adrenergic blocker on CCl_4_ induced hepatic injury and oxidative DNA damage. Histological features (a) and area of necrosis (b) of liver sections stained with hematoxylin and eosin 24 h after CCl_4_ treatment. Typical images were selected from each experimental group (original magnification 200x). The saline + CCl_4_ group showing hepatocellular necrosis; the phentolamine + CCl_4_ group showing mild hepatocellular necrosis. (c) Oxidized DNA in liver was measured 24 hours after exposure to CCl_4_ following pretreatment with saline, phentolamine, or nadolol. Data plotted are mean and SD (*n* = 4 animals in each group).  *∗* denotes significant differences (*p* < 0.05) compared with the saline + CCl_4_ group.  *∗∗∗* denotes significant differences (*p* < 0.001) compared with the saline + CCl_4_ group.

Our findings strongly implicate an important role for the SNS in modulating CCl_4_ induced oxidative stress in liver, based strictly on 6-OHDA ablation of the peripheral SNS. However, it is unclear whether 6-OHDA could affect other cell types, for example, hepatocytes, in mice and through an alternative route directly or indirectly affect oxidative stress or the hepatic antioxidant status. To address this concern, we treated mice with drugs that selectively block *α*- or *β*-adrenergic receptors. Phentolamine, an *α*-blocker, and nadolol, a *β*-blocker, were delivered intraperitoneally over 5 consecutive days before injection with CCl_4_. Nadolol had no significant effect on hepatic injury and antioxidant status (Table 2), indicating that *β*-adrenergic stimulation may not enhance or suppress hepatic oxidative stress responses to CCl_4_. Strikingly, phentolamine enhanced antioxidant status in liver (Table 2, *p* = 0.0238) and suppressed both the extent of hepatic necrosis (11.62 ± 2.76% versus 27.55 ± 12.60%, *p* = 0.0009) (Figure 5) and serum levels of AST (*p* = 0.0478), ALT (*p* = 0.0387), ALP (*p* = 0.0346), and LDH (*p* = 0.0247) (Table 2) following CCl_4_ administration. Pretreatment with phentolamine abrogated the increased level of 8-OHdG in CCl_4_-treated livers (*p* = 0.0193). There was no significant difference in levels of 8-OHdG between the nadolol + CCl_4_ group and saline + CCl_4_ group (Figure 5(c)). The magnitude of the effect was similar to that induced by 6-OHDA treatment, pointing to an important role in SNS activation of *α*-adrenergic receptors in regulating antioxidant status in liver.

## 4. Discussion

In the current study we show that the SNS has major roles in regulating lipid peroxidation, oxidative DNA damage, and proinflammatory cytokine production in the liver associated with CCl_4_ toxicity with chemical sympathectomy essentially preventing the major hepatotoxic effects seen within 24 h of CCl_4_ administration. We also present that *α*-adrenergic signaling is required for control of the oxidative stress in hepatocytes after acute CCl_4_ exposure.

The changes of hepatocyte ultrastructure identified in the current study following CCl_4_ administration are in line with previous observations [26] and are likely to be the result of damage to cellular and organelle membrane structure caused by lipid peroxidation [25, 27]. As chemical sympathectomy prevents these changes it is probable that sympathectomy supports the antioxidant defense system, maintaining membrane integrity. Sympathectomy also attenuated CCl_4_ toxicity characterized by the appearance of dysfunctional hepatic nuclei which arise as a result of enhanced lipid peroxidation [24, 27, 28] further supporting the idea that the mode of action of the beneficial effect of sympathectomy is through reducing oxidative stress. Similarly, the observation that 6-OHDA treatment inhibited oxidative stress-mediated DNA damage in CCl_4_-treated mice suggests that sympathectomy, at least in part, may attenuate oxidative stress-induced DNA damage through the inhibition of lipid peroxidation and the reduction of 4-HNE generation because 4-HNE is biologically reactive and known to cause DNA damage [25, 30].

The cytokine/chemokine array data also demonstrate an inhibitory effect of chemical sympathectomy on CCl_4_ induced hepatic proinflammatory responses. CCl_4_ induced production of inflammation-associated cytokines (IL-1*α*, IL-10, leptin, TIMP-2, and sTNFR I) in the liver, in part a consequence of combined lipid peroxidation [31–33] and hepatocyte necrosis [34], results in an immunostimulatory environment [16, 35, 36]. Presumably the inhibitory effects of sympathectomy on CCl_4_ induced liver cell damage were sufficient to prevent activation of inflammatory cascades. It may also be possible that the SNS has a direct regulatory effect on resident Kupffer [37] and stellate cells [38] within the liver. We also identified an effect of sympathectomy on elevation of hepatic leptin following CCl_4_ induced injury. There is increasing evidence that leptin augments inflammatory and profibrogenic responses to hepatic injury [39, 40] whilst downregulation of leptin decreases liver fibrosis [40, 41]. Sympathectomy appears therefore to potentially have both protective anti-inflammatory and potentially antifibrogenic effects.

CCl_4_ also induced or upregulated the protein expression levels of 5 inflammation-associated chemokines (CCL3, CCL5, CCL9, CCL11, and CXCL11), a response inhibited by pretreatment with 6-OHDA. Increased expression of CCL3 and CCL5 following CCl_4_ treatment is consistent with previous studies [42, 43]. To the best of our knowledge, this is the first demonstration of expression of CCL9, a mouse CC chemokine and strong chemoattractant for bone marrow cells [44], in liver. CCL11 is a known potent inducer of eosinophil chemotaxis and regulates the recruitment to the liver after CC1_4_ induced hepatic injury to facilitate liver regeneration [45]. CXCL11 release has previously been shown to be induced by oxidative stress exposure [46] and liver ischemia/reperfusion injury [47] supporting the idea that the antioxidant effect of sympathectomy is possibly attributable to the downregulation of CXCL11 and possibly the other cytokines and chemokines studied including GM-CSF [48].

Studies describing an interaction with pharmacological sympathetic blockade have been reported for CCl_4_. Pretreatment with either prazosin, an *α*_1_-selective adrenoreceptor antagonist, or yohimbine, an *α*_2_-selective adrenoreceptor antagonist, abolishes methamphetamine potentiation of CCl_4_ hepatotoxicity. However, neither prazosin nor yohimbine has any effects on toxicity produced by CC1_4_ alone [5, 49]. The ability of phenoxybenzamine, a nonselective, irreversible *α*-adrenergic receptor antagonist, to counteract the hepatotoxic effect of CCl_4_ by preventing the action of catecholamines [50], has been reported. A similar observation was noticed in the recent study of the interaction between dopaminergic agonist piribedil and CCl_4_. Administration of piribedil results in amelioration of CCl_4_ induced liver damage probably due to its *α*_2_-adrenoceptor antagonist properties to reduce sympathetic outflow and then decrease the extent of lipid peroxidation [51]. Since both phenoxybenzamine and piribedil cross the blood-brain barrier, they would be expected to antagonize pan *α*- or *α*_2_-adrenoreceptors, respectively, within the central nervous system (CNS). Our findings that phentolamine, a pan *α*-adrenergic antagonist which has no access to the CNS, suppresses hepatic injury through oxidative stress suggest that suppression of antioxidant status by SNS is associated with the peripheral release of catecholamines. Moreover, we show that it is unlikely that the *β*-adrenergic signaling modulates CCl_4_ induced oxidative stress by pretreatment with nadolol. This observation appears to be consistent with a role for isoproterenol, an agonist at both *β*_1_- and *β*_2_-adrenoreceptors, found to have no effect on potentiation of CCl_4_ induced hepatotoxicity [52] and may be due to the concept that *β*-receptor activation decreases H_2_O_2_ synthesis in hepatocyte plasma membrane [53, 54]. Although we favor the idea that the *α*-blocker acts directly on hepatocytes we cannot eliminate the potential contribution of catecholamines on blood flow to liver. Activation of the SNS results in production of both “classical” neurotransmitters norepinephrine and epinephrine and cotransmitters such as adenosine triphosphate and adenosine. Hepatocytes express various adrenergic and purinergic receptors that are sensitive to these molecules, and the production of cytokines/chemokines is probably modulated by activation of these receptors [1, 55] and not necessarily by adrenergic activity alone.

## 5. Conclusions

In conclusion, we found that sympathectomy or *α*-adrenergic blockade decreased hepatic lipid peroxidation in CCl_4_ induced liver injury. Our results also suggest that the SNS may regulate inflammatory cytokine and chemokine production following hepatic injury through regulation of lipid peroxidation. This study demonstrates that modulation of the SNS can potentially influence the outcome of acute liver damage and provides a basis to explore the effects of adrenergic modifiers on treatment of drug-induced liver injury in humans.

## Supplementary Material

Figure S1: Effect of 6-OHDA on hepatic sympathetic innervation. Representative immunohistochemistry of sympathetic tyrosine hydroxylase nerve positive ﬁbers of liver of mice with or without 6-OHDA. Typical images were selected from each experimental group (original magniﬁcation 200×). Scale bar = 100 μm. Arrows indicate sympathetic nerve ﬁbers.
Figure S2: Effect of 6-OHDA on CCl_4_ induced hepatic injury. Histological features (a), necrosis area (b) and severity score (c) of liver sections stained with hematoxylin and eosin 24 h after CCl_4_ treatment. Typical images were selected from each experimental group (original magniﬁcation 40×-400×). The saline + olive oil group and the 6-OHDA + olive oil group showing normal hepatic architecture; the saline + CCl_4_ group showing hepatocellular necrosis; the 6-OHDA + CCl4 group showing mild hepatocellular necrosis. The histological changes were scored in Methods. Data plotted are mean and SD (n = 6 animals in each group). ^###^denotes signiﬁcant differences (p < 0.001) compared with the saline + CCl_4_ group.

## Figures and Tables

**Table 1:** Effect of sympathectomy on CCl_4_ induced lipid peroxidation in liver.

Parameter	Saline + olive oil (*n* = 6)	6-OHDA + olive oil (*n* = 6)	Saline + CCl_4_ (*n* = 6)	6-OHDA + CCl_4_ (*n* = 6)
4-Hydroxy-2-nonenal (4-HNE, *μ*g/*μ*g protein)	0.23 ± 0.02	0.18 ± 0.02^*∗∗*^	0.33 ± 0.07^*∗*^	0.23 ± 0.03^##,††^

The results are presented as mean ± SD.

*∗* denotes significant differences compared with the saline + olive oil group (*p* < 0.05).

*∗∗* denotes significant differences compared with the saline + olive oil group (*p* < 0.01).

## denotes significant differences compared with the saline + CCl_4_ group (*p* < 0.01).

†† denotes significant differences compared with the olive oil + 6-OHDA group (*p* < 0.001).

**Table 2:** Hepatic lipid peroxidation and serum biochemical markers in mice after 24 hours of CCl_4_ treatment with or without pretreatment of *α*-adrenergic blocker or *β*-adrenergic blocker.

Parameter	Saline + CCl_4_ (*n* = 4)	Phentolamine + CCl_4_ (*n* = 4)	Nadolol + CCl_4_ (*n* = 4)
4-Hydroxy-2-nonenal (4-HNE, *μ*g/*μ*g protein)	0.34 ± 0.04	0.25 ± 0.01^*∗*^	0.28 ± 0.02
Aspartate transaminase (AST, IU/L)	16,695 ± 5,203	6,038 ± 3,320^*∗*^	13,571 ± 5,758
Alanine transaminase (ALT, IU/L)	14,559 ± 3,268	7,318 ± 3,799^*∗*^	10,830 ± 4,381
Alkaline phosphatase (ALP, IU/L)	131 ± 14	106 ± 21^*∗*^	140 ± 31
Lactate dehydrogenase (LDH, IU/L)	30,656 ± 9,387	8,994 ± 5,986^*∗*^	17,483 ± 10,584

The results are presented as mean ± SD.

*∗* denotes significant differences compared with the saline + CCl_4_ group (*p* < 0.05).
