# Supplementary material for: Sympathetic Nervous System Control of Carbon Tetrachloride-Induced Oxidative Stress in Liver through α-Adrenergic Signaling
Source: Oxid Med Cell Longev. 2015 Dec 21;2016:3190617. doi: 10.1155/2016/3190617 (PMC4699022; doi:10.1155/2016/3190617)
Supplement: Supplementary file 1 — Figure S1: Effect of 6-OHDA on hepatic sympathetic innervation. Representative immunohistochemistry of sympathetic tyrosine hydroxylase nerve positive ﬁbers of liver of mice with or without 6-OHDA. Typical images were selected from each experimental group (original magniﬁcation 200×). Scale bar = 100 μm. Arrows indicate sympathetic nerve ﬁbers. Figure S2: Effect of 6-OHDA on CCl4 induced hepatic injury. Histological features (a), necrosis area (b) and severity score (c) of liver sections stained with hematoxylin and eosin 24 h after CCl4 treatment. Typical images were selected from each experimental group (original magniﬁcation 40×-400×). The saline + olive oil group and the 6-OHDA + olive oil group showing normal hepatic architecture; the saline + CCl4 group showing hepatocellular necrosis; the 6-OHDA + CCl4 group showing mild hepatocellular necrosis. The histological changes were scored in Methods. Data plotted are mean and SD (n = 6 animals in each group). ###denotes signiﬁcant differences (p < 0.001) compared with the saline + CCl4 group. [file 3190617.f1.pdf]

**Supplementary Material**

Figure S1: Effect of 6-OHDA on hepatic sympathetic innervation. Representative immunohistochemistry of sympathetic tyrosine hydroxylase nerve positive fibers of liver of mice with or without 6-OHDA. Typical images were selected from each experimental group (original magnification 200×). Scale bar = 100 μm. Arrows indicate sympathetic nerve fibers.

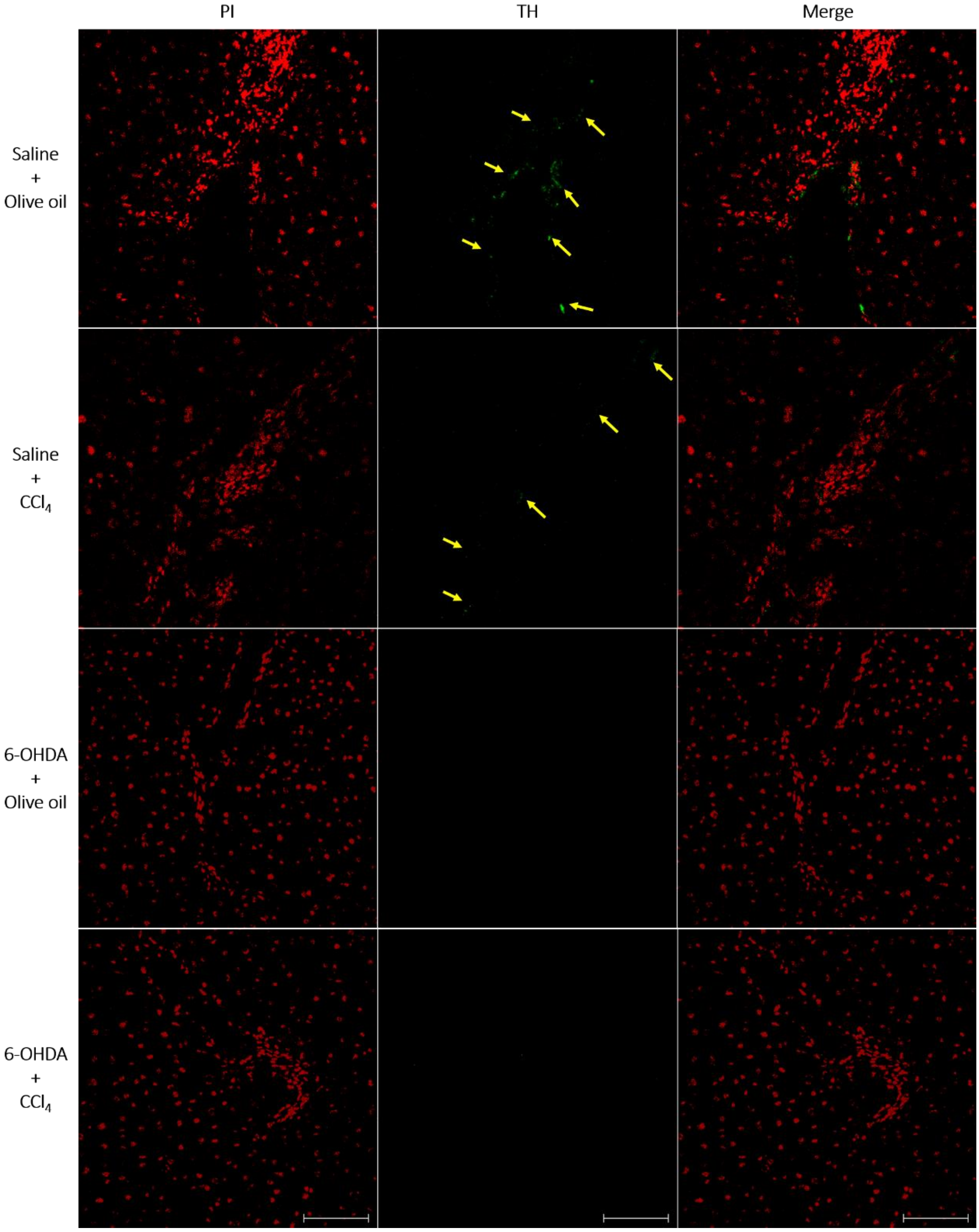

Figure S2: Effect of 6-OHDA on $CCl_4$ induced hepatic injury. Histological features (a), necrosis area (b) and severity score (c) of liver sections stained with hematoxylin and eosin 24 h after $CCl_4$ treatment. Typical images were selected from each experimental group (original magnification 40×-400×). The saline + olive oil group and the 6-OHDA + olive oil group showing normal hepatic architecture; the saline + $CCl_4$ group showing hepatocellular necrosis; the 6-OHDA + $CCl_4$ group showing mild hepatocellular necrosis. The histological changes were scored in Methods. Data plotted are mean and SD (n = 6 animals in each group). ###denotes significant differences ($p < 0.001$) compared with the saline + $CCl_4$ group.

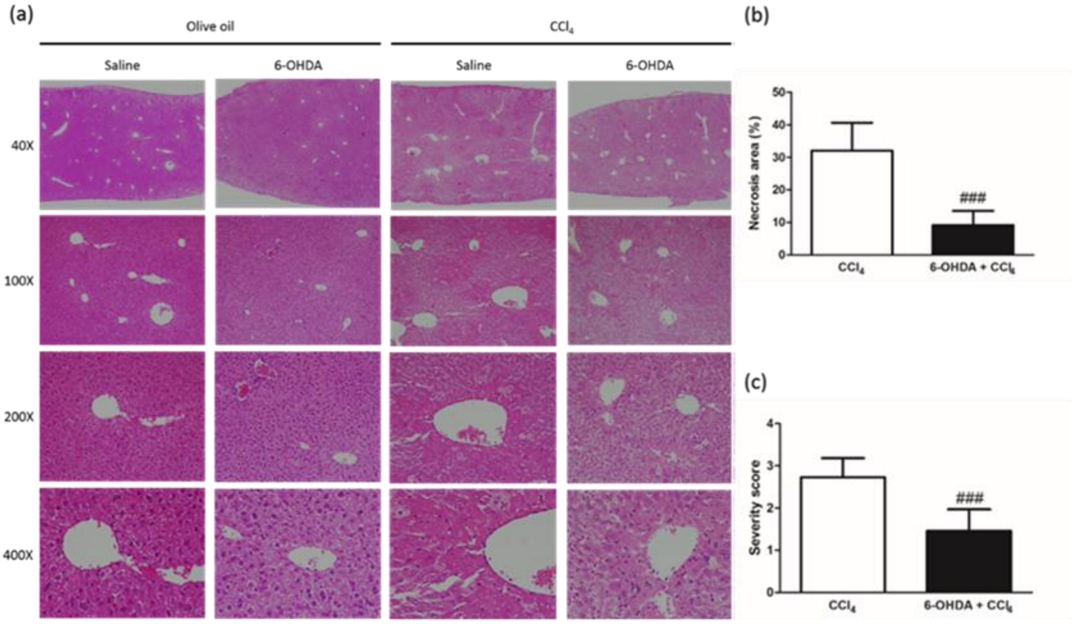

Table S1: Serum biochemical markers in mice after 24 hours of $CCl_4$ treatment with or without 6-OHDA

| Parameter | Saline + Olive oil (n = 6) | 6-OHDA + Olive oil (n = 6) | Saline + $CCl_4$ (n = 6) | 6-OHDA + $CCl_4$ (n = 6) |
|---|---|---|---|---|
| Aspartate transaminase (AST, IU/L) | 134 ± 18 | 115 ± 59 | 17,830 ± 3,078$^{***}$ | 4,115 ± 1,755$^{\#\#\#\#}$ |
| Alanine transaminase (ALT, IU/L) | 40 ± 11 | 51 ± 28 | 15,519 ± 4,678$^{***}$ | 7,809 ± 2,527$^{\#\#}$ |
| Alkaline phosphatase (ALP, IU/L) | 87 ± 22 | 59 ± 23 | 128 ± 11$^{**}$ | 56 ± 26$^{\#\#}$ |
| Lactate dehydrogenase (LDH, IU/L) | 942 ± 483 | 1,166 ± 744 | 28,764 ± 8,063$^{***}$ | 11,139 ± 5,496$^{\#\#\#}$ |

The results are presented as mean ± S.D.

$^{**}$Denotes significant differences compared with the Saline + Olive oil group ($p < 0.01$).

$^{***}$Denotes significant differences compared with the Saline + Olive oil group ($p < 0.001$).

$^{\#\#}$Denotes significant differences compared with the Saline + $CCl_4$ group ($p < 0.01$).

$^{\#\#\#}$Denotes significant differences compared with the Saline + $CCl_4$ group ($p < 0.001$).

$^{\#\#\#\#}$Denotes significant differences compared with the Saline + $CCl_4$ group ($p < 0.0001$).
